# P-261. The Epidemiological Impact of Mycobacterium tuberculosis and HIV Co-Infection: A Global and Local Perspective

**DOI:** 10.1093/ofid/ofaf695.482

**Published:** 2026-01-11

**Authors:** Aliyu Evuti Haruna, Nma Bida Alhaji

**Affiliations:** Africa Center of Excellence for Mycotoxin and Food Safety, Federal University of Technology, Minna,Niger State, Minna, Niger, Nigeria; Africa Center of Excellence for Mycotoxin and Food Safety, Federal University of Technology, Minna,Niger State, Minna, Niger, Nigeria

## Abstract

**Background:**

The co-infection of *Mycobacterium tuberculosis* (TB) and HIV poses a major global health threat, particularly in regions with high HIV prevalence. An estimated 4.4 million people are co-infected, with TB being a leading cause of AIDS-related deaths. While over 2 billion people worldwide carry latent TB, HIV greatly increases the risk of reactivation from 5–10% over a lifetime to 5–10% annually. HIV also raises the risk of primary TB infection by 2 to 5 times and is linked to extra-pulmonary TB in over 70% of cases. This co-infection significantly worsens disease outcomes and complicates management.

**Methods:**

A comprehensive review of peer-reviewed literature and epidemiological reports was conducted to assess the prevalence, interaction, and impact of TB-HIV co-infection. Data were extracted from WHO surveillance documents and region-specific studies, including those from Niger State, Nigeria. Comparative analysis was performed using descriptive statistics and prevalence ratios to estimate risk differentials and co-infection burden. Studies selected met inclusion criteria based on sample size ( >100 participants)

**Results:**

Findings reveal a high burden of TB among HIV-infected individuals, with reactivation rates up to 10 times higher than in HIV-negative populations. Extrapulmonary TB is notably more prevalent in HIV-positive patients (70% vs. < 20%). In Niger State, co-infection rates surpass national averages, leading to increased primary infections and worse health outcomes. TB also accelerates HIV progression by boosting viral replication. However, integrated treatment approaches in high-burden areas have been shown to effectively reduce morbidity.

**Conclusion:**

The interplay between HIV and TB creates a synergistic burden that significantly undermines global health efforts. The co-infection not only increases morbidity and mortality but also complicates treatment due to overlapping symptoms and drug interactions. Enhanced epidemiological surveillance, integrated management programs, and targeted interventions in high-burden regions like Niger State are essential. Public health policies must prioritize co-infection screening, early diagnosis, and dual therapy to mitigate the escalating global impact of HIV-TB co-infection.

**Disclosures:**

All Authors: No reported disclosures

